# Overcoming Chimeric Antigen Receptor (CAR) Modified T-Cell Therapy Limitations in Multiple Myeloma

**DOI:** 10.3389/fimmu.2020.01128

**Published:** 2020-06-05

**Authors:** Estefanía García-Guerrero, Belén Sierro-Martínez, Jose Antonio Pérez-Simón

**Affiliations:** Instituto de Biomedicina de Sevilla, UGC de Hematología, Hospital Universitario Virgen del Rocío and Consejo Superior de Investigaciones Científicas (CSIC) and Centro de Investigación Biomédica en Red Cáncer (CIBERONC), Universidad de Sevilla, Seville, Spain

**Keywords:** CAR T-cell, myeloma, toxicities, antigen escape, soluble protein, allogeneic CAR T-cell

## Abstract

Multiple myeloma (MM) remains an incurable disease regardless of recent advances in the field. Therefore, a substantial unmet need exists to treat patients with relapsed/refractory myeloma. The use of novel agents such as daratumumab, elotuzumab, carfilzomib, or pomalidomide, among others, usually cannot completely eradicate myeloma cells. Although these new drugs have had a significant impact on the prognosis of MM patients, the vast majority ultimately become refractory or can no longer be treated due to toxicity of prior treatment, and thus succumb to the disease. Cellular therapies represent a novel approach with a unique mechanism of action against myeloma with the potential to defeat drug resistance and achieve long-term remissions. Genetic modification of cells to express a novel receptor with tumor antigen specificity is currently being explored in myeloma. Chimeric antigen receptor gene-modified T-cells (CAR T-cells) have shown to be the most promising approach so far. CAR T-cells have shown to induce durable complete remissions in other advanced hematologic malignancies like acute lymphocytic leukemia (ALL) and diffuse large B-cell lymphoma (DLBCL). With this background, significant efforts are underway to develop CAR-based therapies for MM. Currently, several antigen targets, including CD138, CD19, immunoglobulin kappa (Ig-Kappa) and B-cell maturation antigen (BCMA), are being used in clinical trials to treat myeloma patients. Some of these trials have shown promising results, especially in terms of response rates. However, the absence of a plateau is observed in most studies which correlates with the absence of durable remissions. Therefore, several potential limitations such as lack of effectiveness, off-tumor toxicities, and antigen loss or interference with soluble proteins could hamper the efficacy of CAR T-cells in myeloma. In this review, we will focus on clinical outcomes reported with CAR T-cells in myeloma, as well as on CAR T-cell limitations and how to overcome them with next generation of CAR T-cells.

## Introduction

Multiple myeloma (MM) is an hematological malignancy characterized by the clonal proliferation of malignant plasma cells (1). Myeloma develops from a pre-malignant monoclonal proliferation of plasma cells (monoclonal gammopathy of undetermined significance) which progresses to smoldering myeloma and finally to symptomatic disease (1, 2). With an incidence of 5.6 cases per 100.000 people/year in Western countries it accounts for 1% of all cancers and around 10% of hematological malignancies (3). Diagnosis of MM is based on the presence of clonal plasma cells plus monoclonal protein in serum or urine and clinical manifestations including hyper*c*alcemia, *r*enal impairment, *a*nemia and/or *b*one lesions (acronym: CRAB) (4, 5). Levels of albumin, β2microglobulin and LDH together with the presence or not of high risk cytogenetic abnormalities, including del(17p), and/or t(4;14) and/or t(14;16), allows to identify subgroups of patients with very different outcomes varying from 82% overall survival (OS) at 5 years for the low risk, 62% for the intermediate risk and 40% for the high risk subgroups (6).

Great advances have been achieved in the last decade in the treatment of MM with the discovery of new therapeutic agents such as immunomodulatory drugs (thalidomide, lenalidomide, pomalidomide), proteasome inhibitors (bortezomib, carfilzomib), monoclonal antibodies (daratumumab and elotuzumab), and the use of hematopoietic stem cell transplantation (1, 4, 7). However, MM remains an incurable disease as patients almost invariably relapse upon treatment and the probabilities to obtain disease response decrease after each relapse and the time to progression does shorten in every relapse. It is therefore necessary to develop more efficient MM therapies (8).

Engineered T-cells expressing chimeric antigen receptors (CARs) have demonstrated encouraging results in the treatment of relapsed/refractory hematological malignancies (9–12). CARs are synthetic receptor proteins integrated by an extracellular antigen-binding domain derived from a single-chain variable fragment (scFv) of a monoclonal antibody linked to a T cell receptor (TCR)-derived CD3ζ chain, subsequently redirecting cytolytic T-lymphocytes to cells expressing this specific antigen in a human leukocyte antigen (HLA)-independent manner (3, 13). Second- and third-generation CARs present further costimulatory domains such as CD28, 4-1BB, or OX40 to potentiate T-cell activation. Fourth-generation CAR T-cells may include controllable on-off switch proteins, a suicide gene or molecules to potentiate T-cell function, expansion and reduce exhaustion (3, 14).

Two anti-CD19 CAR T-cell products, Tisagenlecleucel (Kymriah) and Axicabtagene Ciloleucel (Yescarta), have been approved by US Food and Drug Administration (FDA) and the European Medicines Agency (EMA) for the treatment of acute lymphoblastic leukemia (ALL) and diffuse large B cell lymphoma (DLBCL). Sustained durable complete remissions have been accomplished with CD19 CAR T-cell products in relapsed/refractory ALL patients which have prompted attention to a possible alternative to overcome actual treatment limitations in MM (10, 12).

As far as MM is concerned, numerous CAR T-cell products are under development. B-cell maturation antigen (BCMA) is the predominantly used target against MM based on its high expression in the surface of malignant plasma cells and restricted expression in normal tissues/cells except for a low-level expression in mature B-cells. BCMA is vital for the survival and proliferation of MM cells, it is expressed in most MM patient samples (60–100%) and its efficacy as a MM antigen for targeted immunotherapy has been tested in several clinical trials. Other targets under development include CD38, CD138, CD19, or immunoglobulin kappa light chain (Ig-Kappa) (3, 15, 16). Despite the promising results achieved by CAR T-cell administration in MM in terms of response rates, the absence of a plateau corresponding with the absence of durable remissions is common to all studies. Clinical experience with CAR T-cell therapy has pointed out several limitations of this technology such as lack of effectiveness, toxicities, antigen loss, interference with soluble proteins or manufacturing issues (15, 17).

In this review, we will report clinical outcomes achieved so far with CAR T-cells for the treatment of MM, as well as focus on their limitations and how to overcome these restrictions with next generation CAR T-cells.

## Car T-Cells in Clinical Trials for MM

Selection of a suitable antigen is essential for the development of an optimal CAR T-cell product. As the recognition of an antigen by the CAR is HLA-independent, the target must be expressed in the cell surface. Besides, the antigen must be homogeneously expressed in tumor cells and have an essential role in their proliferation and survival to avoid escape from CAR T-cells recognition. It is also essential that the chosen antigen is not expressed in vital healthy tissues to avoid undesired on-target, off-tumor toxicities (15). Although BCMA is the predominantly used target for CAR T-cell products for the treatment of MM, several other antigens have been studied and some are being evaluated in clinical trials.

## CD38

CD38 is a transmembrane glycoprotein implicated in calcium regulation, signal transduction and cell adhesion. Among the hematological cell lineages, CD38 is highly expressed on precursor B-cells, plasma cells, NK cells and myeloid precursors. CD38 is also expressed on gut, prostate cells, pancreas, nervous system, muscle cells and osteoclasts (18). Since the FDA approval of several anti-CD38 monoclonal antibodies for the treatment of MM in 2015 (daratumumab, istuximab) (19, 20) the generation of CD38 CAR T-cells has been extensively studied preclinically (21). The wide expression of CD38 among hematopoietic cells might be a critical inconvenient for its clinical application due to possible on-target, off-tumor toxicities. To date CD38 CAR T-cells are under clinical investigation in several trials. In the study NCT03464916, a CD38 CAR T-cell product is being used as monotherapy for relapsed/refractory myeloma to evaluate its efficacy and safety although no outcomes have been posted yet. Moreover, CD38 is also being evaluated within clinical trials in combination with other target antigens including a dual specificity CD38 and BCMA CAR T-cell product (NCT03767751), and a combination CAR T therapy with CD19 (NCT03125577). Other approaches targeting several MM antigens including CD38, BCMA, CD138 and CD56 are being explored (NCT03271632, NCT03473496). A fourth generation CAR T-cell product targeting multiple antigens, including CD38, and expressing simultaneously interleukin-7 (IL-7) and chemokine (C-C motif) ligand 19 (CCL19), is also under clinical investigation for the treatment of relapsed/refractory MM patients (NCT03778346). Nevertheless, results from these CD38-targeted CAR T therapies have not been published to date.

## CD138

CD138 belongs to the syndecan family type I transmembrane proteoglycans and it is implicated in wound healing, cell adhesion and endocytosis. CD138 is expressed on the surface of mature epithelial cells, nonetheless its expression is restricted within the hematopoietic system to normal and tumor plasma cells (22). It has been correlated with survival and disease progression of MM, and its inhibition promotes apoptosis of myeloma cells (23, 24). CD138 has been proven to be an effective target antigen for the treatment of MM in preclinical studies (25). To date, there is only one published clinical trial for the study of autologous CD138 CAR T-cells in relapsed/refractory MM patients pretreated with chemotherapy and stem cell transplantation. Five patients were treated with a single average dose of 0.756 × 10^7^ cells/kg of CD138 CAR T-cells. The CAR gene was detectable in peripheral blood in all patients and high levels were persistent for at least 4 weeks after infusion. No severe toxicities were observed apart from infusion-related fever (grade 3) and nausea and vomiting (grade 2). Four patients experienced myeloma regression after CD138 CAR T-cells infusion for 3–7 months, while the other patient progressed despite the presence of CAR in bone marrow until day 90 post-infusion. No complete responses (CR) were achieved in this clinical trial (26) (Tables 1, 2).

**Table 1:** Characteristics of T-cell products generated in each clinical trial.

**Target**	**Identifier (ref)**	**Costimulatory domain**	**Selection of PBMCs from apheresis**	**Expansion cytokines**	**Transfer method**	**% Transduction efficiency (means)**
CD138	NCT01886976 (26)	4-1BB	No selection	IFN-Y + IL-2	Lentiviral vector	32
CD19	NCT02135406 (27)	4-1BB	No selection	NR	Lentiviral vector	10.1
kappaLC	NCT00881920 (28)	CD28	No selection	IL-2/IL-7 + IL-15	Retroviral vector	82 (IL-2)/ 89 (IL-7 + IL-15)
BCMA	NCT02215967 (29)	CD28	No selection	IL-2	Retroviral vector	44.38
BCMA	NCT02546167 (30)	4-1BB	NR	IL-2	Lentiviral vector	17.47
BCMA	NCT02658929 (31)	4-1BB	No selection	IL-2	Lentiviral vector	85 CD4+ (42–98) 13 CD8+ (2–47)
BCMA	NCT03090659 (32)	CD28	T cell selection	IL-2	Lentiviral vector	NR
BCMA	NCT03430011 (33)	4-1BB	NR	NR	Lentiviral vector	NR
BCMA	NA (34)	4-1BB	NR	NR	Retroviral vector	NR
BCMA	NCT03338972 (35)	4-1BB	Positive selection CD4/CD8	NR	Lentiviral vector	NR
BCMA	NCT03288493 (36)	4-1BB	NR	NR	Transposon-based piggy- Bac system	NR
BCMA	NCT03274219 (37, 38)	4-1BB	No selection	IL-2, PI3K inhibitor	Lentiviral vector	NR

*PBMCs, peripheral blood mononuclear cells; INF-γ, interferon gamma; IL, interleukin; NR, not reported; NA, non-applicable; PI3K, phosphoinositide 3 kinase*.

**Table 2:** Published clinical trials of CAR T-cell therapy in multiple myeloma.

**Target**	**Identifier (ref)**	**Phase**	***N***	**Pre-conditioning regimen**	**CAR- T dosage (cells/kg)**	**Prior treatments (mean)**	**Median follow-up (months)**	**Side effects**	**Clinical effects**	**Progression-free survival**	**BCMA^**−**^ relapse**
CD138	NCT01886976 (26)	1/2	5	PCD/CP/VAD	0.756 × 10^7^ (median)	10	NR	80% fever (G3)	SD (4) PD (1)	NR	NA
CD19	NCT02135406 (27)	1	10	Mel + ASCT	1–5 × 10^7^	6	NR	CRS (G1) (1) Intestinal GVHD (1) Mucositis (1)	sCR (1) VGPR (6) PR (2)	200.8 days	NA
kappaLC	NCT00881920 (28)	1	7	Cy (4) or none (3)	0.92 × −1.9 × 10^8^ cells/m^2^	4	NR	Lymphopenia (G3) (1) No CRS	SD (4) NR (3)	NA	NA
BCMA	NCT02215967 (29)	1	24	Cy + Flu	0.3 × −9 × 10^6^	9.5	NR	38% CRS (grade 3–4) 44% CRS (grade 1–2) Neurotoxicity (1)	81% ORR sCR (2) VGPR (9) PR (4)	31 weeks	1 BCMA^−^progression
BCMA	NCT02546167 (30)	1	25	Cy or none	1–5 × 10^7^ or 1–5 × 10^8^	7	NR	88% CRS (G ≥ 3: 8 patients) 32% neurotoxicity	ORR (48%) cohort 1 (44%), cohort 2 (20%), cohort 3 (64%)	65, 57, 125 days (cohort 1, 2, or 3)	No BCMA- clones found
BCMA	NCT02658929 (31)	1	33	Cy + Flu	50 × 150 × 450 × and 800 × 10^6^	7-8	11.3	70% CRS (grade 1–2) 6% CRS (grade 3) 42% neurotoxicity	ORR (85%), ≥ CR (45%) sCR (36%)	11.8 months	NR
BCMA	NCT03090659 (32)	1	57	Cy	0.07–2.1 × 10^6^	3	12	83% CRS (grade 1–2) 7% CRS (grade 3) Neurotoxicity (grade 1) (1)	88% ORR (68% CR 5% VGPR 14% PR)	15 mo (<40% BCMA^+^) 11 mo (>40% BCMA^+^)	NR
BCMA	NCT03430011 (33)	1/2	44	Cy + Flu	50 × or 150 × 10^6^	7	2.6	80% CRS (G≥3 9%) 25% neurotoxicity (G≥3 7%)	82% ORR (27% CR)	NA	No relapses reported
BCMA	NA (34)	1	11	Cy or Flu + Cy	72 × 137 × 475 × 818 × 10^6^	6	NR	40% CRS (G1-2) 20% CRS (G3) 10% neurotoxicity (G2)	64% ORR	NA	No relapses reported
BCMA	NCT03338972 (35)	1	7	Cy + Flu	5 × or 15 × 10^7^	8	3.7	86% CRS (G ≤ 2) No neurotoxicity	100% ORR	NA	1 BCMA^−^ relapse
BCMA	NCT03288493 (36)	1/2	23	Cy + Flu	0.75 × −15 × 10^6^	6	137 days	9,5% CRS (G1-2) 4.8% neurotoxicity (G2)	63% ORR	NA	NR
BCMA	NCT03274219 (37, 38)	1	22	Cy + Flu	150 × 450 × 800 × 1200 × 10^6^	7	23 weeks	59% CRS (5G1, 7G2, 1G3) 23% neurotoxicity (1G1, 2G2, 1G3, 1G4)	83% ORR	NR	NA

*PCD, pomalidomide-cyclophosphamide-dexamethasone; CP, chlorambucil-prednisone; VAD, vincristine-doxorubicin-dexamethasone; PD, partial disease; Cy, cyclophosphamide; Flu, fludarabine; G, grade; Mel, melphalan; ASCT, autologous stem cell transplantation; NR, not reported; NA, non-applicable*.

## CD19

CD19 is a B lymphocyte-specific surface protein which constitutes a component of the B-cell co-receptor complex and belongs to the immunoglobulin superfamily. It is expressed throughout B-cell differentiation, from pre- to mature B-cells (39, 40). Although expression of CD19 is rare in plasma cells, there is a small population of CD19_positive_ myeloma cells which has been discovered to be more pre-mature and might constitute the myeloma-initiating or myeloma-stem cells. They have been associated with high-risk disease, poor prognosis, relapses and reduced survival (41, 42). CD19 is the most widely studied target antigen for the development of CAR T therapies with two products (Kymriah and Yescarta) approved for the treatment of ALL and DLBCL, and multiple published and ongoing clinical trials (43). Therefore, targeting CD19 in MM represents an interesting strategy to focus on this CD19_positive_ myeloma cell subset. In the study NCT02135406, 10 refractory MM patients were infused with autologous CD19 CAR T-cells after autologous stem cell transplantation (ASCT). All patients included received previously a first ASCT resulting in poor response with progression-free survival (PFS) of <1 year. CD19 expression in myeloma cells was assessed by flow cytometry and, as expected, the predominant myeloma population was CD19_negative_ in all patients. However, seven out of nine evaluable patients presented a small CD19_positive_ subset (from 0.04 to 1.6%) (27). Patients were infused with 1–5 x 10^7^ cells/kg CD19 CAR T-cells (CTL019) 2 weeks after high-dose melphalan and a second ASCT. In 2015, the clinical outcome of the first treated patient was reported with a sustained complete remission lasting for at least 12 months in spite of CD19 expression-absence in most of the myeloma cells (44). Six out of 10 patients infused obtained a very good partial response (VGPR) at day 100 post-transplantation. To find out whether CTL019 infusion did increase PFS after ASCT, they compared PFS from each subject after prior ASCT alone vs. ASCT+CTL019 treatment. Two patients significantly increased PFS after CTL019 treatment (479 vs. 181 days; 249 vs. 127 days). These results highlight the recognition of target antigen by the CAR even when it is present in very low intensity or non-detectable by flow cytometry (45) (Tables 1, 2). The same group conducted a phase II clinical trial (NCT02794246) to study the efficacy of CD19 CAR T-cells infusion 60 days post-ASCT in 5 MM patients. No results have been published yet. The combination of autologous/allogenic CD19 CAR T-cells and BCMA CAR T-cells has also been explored.

### Immunoglobulin Kappa Light Chain

Despite the success achieved with CD19 CAR T-cells in hematological malignancies, sustained clinical responses need long-term *in vivo* CAR persistence which is linked to B-cell aplasia and therefore impaired humoral immunity. This toxicity occurs due to the expression of CD19 in normal B-lymphocytes as it is a pan-B-cell expression marker. New antigens with more restricted distribution need to be explored to reduce cytotoxicity and allow normal humoral immunity recovery even with *in vivo* CAR persistence (10, 28). Expression of surface immunoglobulin, with either kappa (κ) or lambda (λ) light chain, is limited to mature B-cells and mature B-cell malignancies. Although normal plasma cells do not maintain immunoglobulin expression, a clonogenic MM-initiating population has been described which expresses surface immunoglobulin (46). Directing CAR T-cells to a certain type of immunoglobulin light chain (κ or λ) would eliminate the MM-monoclonal cells expressing the target light chain while avoiding cytotoxicity against normal mature B-cells expressing the remaining one. Therefore, targeting immunoglobulin kappa light chain (IgkLC) might be a feasible strategy to direct CAR T therapy to MM while being more restrained within the whole B-cell subset. In Ramos et al. (28) a clinical trial (NCT00881920) is described to evaluate safety and efficacy of κCAR T-cells in chronic lymphocytic leukemia (CLL), non-Hodgkin lymphoma (NHL) and MM patients. Seven relapsed/refractory MM patients were infused with 0.92–1.9 × 10^8^ cells/m^2^ after cyclophosphamide preconditioning. No serious CAR-related adverse events were reported excluding a patient with grade 3 lymphopenia. According to clinical responses, four out of seven patients reached stable disease (SD) from 6 to 24 months. The other three patients did not respond to the therapy (28) (Tables 1, 2).

### B-Cell Maturation Antigen

B-cell maturation antigen is the ultimate target studied for the development of CAR T therapies for MM with up to 53 clinical trials worldwide. BCMA is a transmembrane glycoprotein which constitutes part of the tumor necrosis factor receptor (TNFR) superfamily. It participates in the B-cell differentiation into plasma cells and in its long-term survival and proliferation (16, 47). Besides, expression of BCMA was confirmed by Friedman et al. (48) in malignant MM cells in 100% of the patients analyzed, though levels were variable. Several clinical trials have assessed the benefits of targeting BCMA for the treatment of MM either with anti-BCMA bispecific T-cell engagers (BiTE) or anti-BCMA antibody-drug conjugates (49, 50). Indeed, multiple clinical trials have explored the effect of BCMA CAR T-cells in the treatment of MM (51).

The first clinical trial designed with anti-BCMA CAR T-cells was carried out in the National Cancer Institute (NCT02215967) and results were presented by Brudno et al. (29) in 2018. They enrolled 24 patients, 10 in a dose escalation phase (0.3 × −3 x 10^6^ cells/kg) and 16 were infused with the highest dose (9 × 10^6^ cells/kg). They reported an overall response rate (ORR) of 81% among the 16 patients treated with the highest dose with 2 patients achieving a stringent complete response (sCR), 8 a VGPR, 3 a partial response (PR) and 3 non-responding to treatment. Peak CAR+ cell levels in peripheral blood, occurring 7 days-post-infusion, were associated with anti-myeloma responses. Cytokine release syndrome (CRS), resulting from T-cell activation after CAR T engagement, and neurotoxicity are the major CAR T-related adverse events described to date (52). CRS grade 3–4 was reported in 5 out of 16 patients infused with the highest dose (38%) and mild CRS was present in 7 patients (44%). Neurotoxicity was not reported or limited to delirium or confusion except for patient 15 who presented encephalopathy. Higher levels of bone marrow plasma cells were also associated with a more severe CRS. BCMA expression was also assessed in myeloma cells pre- and post-treatment and patient 11 was found to have BCMA_negative_ myeloma cells at week 56 post-infusion followed by myeloma progression at week 68 with mixed BCMA expression (29) (Tables 1, 2).

Cohen et al. (30) reported a phase I clinical trial (NCT02546167) to evaluate safety and efficacy of BCMA CAR T-cells in relapsed/refractory MM patients. Three different cohorts were studied, cohort 1: 1–5 × 10^8^ BCMA CAR T-cells/kg infused (9 patients), cohort 2: 1–5 × 10^7^ cells/kg + Cyclophosphamide as preconditioning (5 patients), and cohort 3: 1–5 × 10^8^ cells/kg + Cyclophosphamide as preconditioning (11 patients). In this trial, all 25 patients were infused in a 3-dose-split protocol over 3 days (30). CRS was reported in 22 out of 25 patients (88%) (grade 3–4) and neurotoxicity was observed in 8 out of 25 patients (32%). Three patients presented severe neurotoxicity (grade 3–4) which correlated with high tumor burden, a dose of 5 × 10^8^ cells/kg and grade 3-4 CRS. The objective responses within the cohorts were: 44% in cohort 1, 20% in cohort 2 and 64% in cohort 3 (1 CR, 5 VGPR and 1sCR). The ORR was 48% (12 out of 25), with 1-5 x 10^8^ cells/kg being the most effective dose (11 responding-patients) (30) (Tables 1, 2).

In the Bluebird study NCT02658929, 33 patients with relapsed/refractory MM were treated with anti-BCMA CAR T-cells (bb2121) in a dose/escalation study (50–800 × 10^6^ cells/kg) (31). In this study, 25 patients presented CRS, among them 23 (70%) had grade 1–2 and 2 (6%) had grade 3–4. Neurologic toxicities were also remarkable occurring in 14 patients (42%). The ORR was 85% with 15 complete responses (45%), and all responding-patients had negative minimal residual disease (MRD). However, six out of the 15 patients with CR finally relapsed (31) (Tables 1, 2).

In Zhao et al. (32) the authors developed a BCMA-directed CAR T-cell containing two heavy chain-only antibodies (VHH) which targets two different BCMA epitopes (LCAR-B38M). The safety and efficacy of this CAR T-cell product were studied in a clinical trial with 57 patients enrolled (NCT03090659) (32). They reported 51 (90%) patients who developed CRS on variable grades (Table 2). One patient presented neurotoxicity grade 1 with seizure-like activity, agitation and aphasia. The authors described an ORR of 88% with 68% CR, 5% VGPR and 14% PR. They analyzed BCMA expression on all patients but no correlation among BCMA expression, progression-free survival, overall survival or clinical response was found. Correlation between CAR T-cell dose and clinical response was not found either (32) (Tables 1, 2).

The Memorial Sloan Kettering Cancer Center (MSKCC) has developed different CAR T-cell products containing human-derived or fully human scFv antibodies against BCMA named JCARH125, MCARH171, and FCARH143. All CAR T-cell products have been included in phase 1 clinical trials. JCARH125 is an anti-BCMA CAR T-cell product studied in a multicenter clinical trial in the United States (EVOLVE, NCT03430011). In this study, 44 patients were infused with two different dose levels (50 × 10^6^ or 150 × 10^6^ cells/kg). With a 2.6 months median follow-up, 82% overall responses were reported with 27% CR. CRS was present in 80% of patients and neurotoxicity in 25% (33, 51) (Tables 1, 2). MCARH171 expresses a different human-derived scFv antibody than JCARH125 and T-cells were transduced with γ-retrovirus instead of using a lentiviral vector. Safety and efficacy were evaluated in a cohort of 11 patients with an ORR of 64%. CRS grade 1–2 occurred in 40% of patients and 20% had CRS grade 3. Neurotoxicity was only experienced by one patient with grade 1 encephalopathy (34) (Tables 1, 2). It has been described that defined ratios of CD4+:CD8+ in the T-cell product might benefit expansion and function (53). FCARH143 employs the same construction as JCARH125 but CD4^+^ and CD8^+^ T-cells were cultured separately *ex vivo* and infused in a defined 1:1 ratio. Seven patients have been reported to date with an ORR of 100% at 28 days post-infusion. CAR^+^ T-cells were detectable 90 days after infusion. One patient relapsed at day 60 and tumor biopsy demonstrated the presence of a BCMA_negative_ plasma cell population (35) (Tables 1, 2).

A novel CAR T-cell product designed with a non scFv antibody but a different BCMA-specific antibody-mimetic binding domain has been evaluated in a clinical trial. Besides, a different non-viral transfection method was used, named transposon piggy-bac system, which allows more cargo capacity and preferential transfection of stem cell memory T-cells with a lower cost (Table 1). To date, 23 patients have been treated in 5 different dose groups (Table 2). Two patients experienced CRS (grade 1–2) (9.5%) and neurotoxicity was reported in one patient (grade 3) (4.8%). ORR goes from 50% to 100% depending on dose group with a median of 63% in all patients (36, 51) (Table 2).

In Shah et al. (37) the authors designed a clinical trial with a next-generation CAR T-cell (bb21217) using the same construct as bb2121 (31) but with a novel approach by employing phosphoinositide 3 kinase (PI3K) inhibitor bb007 during *ex vivo* expansion to enrich the product in memory-like T-cells (Table 1). In the update presented at the American Society of Hematology Annual Meeting 2019, they reported 22 infused patients with an ORR of 83% (15/18 evaluable patients). CRS occurred in 59% of patients and neurotoxicity in 23% (38) (Table 2).

## Car T-Cell Therapy Limitations in MM

With an increasing number of CAR T-cell-treated patients, observations of therapy-related toxicity and disease relapse are showing the current limitations of this therapeutic modality (17, 54, 55). Key challenges related to CAR T-cell therapy include toxicities, antigen escape, suboptimal activation and persistence of CAR T-cells.

### On/Off-Tumor Toxicity: Treatment-Related Toxicities

Immunotherapy with adoptive T-cells targeting myeloma-associated antigens are at various stages of development and have brought a new hope for cure (31, 38, 56, 57). Nevertheless, severe toxicities accompany this promising technology as it has been reported with the increasing clinical experience with CAR T-cell therapy (58–63). CAR T-cell related toxicities can be divided into two categories: (1) general toxicities due to T-cell recognition and activation against tumor cells and followed by uncontrolled release of high levels of cytokines (on-target, on-tumor toxicities); and (2) toxicities appearing from specific binding between CAR T-cell and its target antigen expressed in normal cells (on-target, off-tumor toxicities) (17, 64).

#### On-Target, On-Tumor Toxicity

Severe and sometimes lethal increases in systemic cytokine levels have been observed in patients treated with CAR T-cells in many clinical trials. Robust interactions between CAR modified T-cells either with tumor or host immune cells may result in CAR T-cell activation and expansion. In some cases, this immune cell activation and uncontrolled cytokine release can be toxic for patients (64). Typically observed CAR T-cell related toxicities are cytokine release syndrome (CRS) and neurotoxicity. CRS is the most-common toxicity of cellular immunotherapy and it appears as a consequence of accelerated expansion and activation of CAR T-cells. This pronounced activation provokes an extreme release of serum levels of interferon gamma, interleukin 6, among other inflammatory cytokines. CRS commonly appears within the first 2 weeks post-infusion of CAR T-cells. Clinical symptoms of this on-target, on-tumor toxicity goes from mild fevers, malaise and flu-like symptoms to severe sepsis-like indications such as high-grade fevers, hypotension, hypoxemia, organ dysfunction, coagulopathy, and pancytopenia which may require intensive care admission (64–66). Treatment with corticosteroids could reduce CRS but to the detriment of effectiveness of CAR T-cells due to suppression of T-cell function and/or induction of T-cell apoptosis (67, 68). Interleukin 6 blockade by anti-interleukin 6 receptor antibodies, such as tocilizumab, is the most commonly used treatment of severe CRS. Besides, tocilizumab does not seem to have a major impact on CAR T-cell efficacy (64–66). Another common adverse event of CAR T-cells is neurotoxicity. This event is observed in up to 42% of patients receiving anti-BCMA CAR T-cells and usually overlaps or appears shortly after CRS (Table 2). Neurotoxicity is commonly restricted to low grade symptoms such as few days of mild confusion, somnolence, and/or word-finding difficulties. Eventually, neurotoxicity can evolve to more severe immune effector cell-associated neurotoxicity syndrome (ICANS) (66) which includes focal deficits, seizures, and fatal cerebral edema (69). Pathophysiology of neurotoxicity is not well-described, but high levels of inflammatory cytokines within central nervous system associated with an increase in endothelial cell activation and vascular permeability have been observed (65). Some factors associated with a higher risk of neurotoxicity include high tumor burden, and more rapid and severe CRS; however, more studies are needed to better understand this toxicity (69).

#### On-Target, Off-Tumor Toxicity

CAR T-cell infusion can also be associated to on-target, off-tumor toxicities due to their uncontrolled growth and excess cytokine release after recognition of target antigen expressed on non-malignant cells (58, 70, 71). The challenge to find tumor-restricted antigens that are not expressed on normal cells, is the major concern in CAR T-cell development (51, 72). Current target antigens of CAR T-cells are usually present on the surface of non-malignant cells, even though at low level. However, this low level could eventually lead to severe off-tumor toxicities (73). B-cell aplasia after CD19 CAR T-cells infusion, is an excellent example of on-target, off-tumor toxicity. The functionality of CD19 CAR T-cells is always followed by B-cell aplasia as a result of the depletion of CD19_positive_ B-cell progenitors. As a consequence, reduced immunoglobulin level is observed in serum of these patients (74). However, while this side effect can be addressed by immunoglobulin replacement (27, 75, 76), damage to more essential organs or tissues can be fatal (61, 73). Among MM target antigens, CD38 has been reported to have medium-expression level on normal hematopoietic cells and non-hematopoietic tissues including prostate epithelial cells. The potency of CAR T-cells does not guarantee safety when targeting this widely expressed protein despite the currently use of anti-CD38 monoclonal antibodies clinically approved (77). Moreover, expression of CD38 on activated T-cells may suppose a detrimental effect because of fratricide cytotoxicity among CD38 CAR T-cells (78). Therefore, the affinity of the scFv antibody used to develop CD38 CAR T-cell product, needs to be accurately optimized to avoid on-target, off-tumor side effects (21, 71). The same happens with CD138 target antigen. Its widely expression leads to concerns with regard to off-tumor toxicities using CD138 CAR T-cells in myeloma patients. Although no severe epithelial toxicities have been noted at present (25), more studies evaluating CD138 CAR T-cells should include strategies to avoid off-tumor toxicities while maintaining anti-tumor effect. BCMA is the most commonly targeted antigen in CAR T-cell therapies for MM. BCMA plays a fundamental role in long-term plasma cell survival and B-cell differentiation into plasma cells (47) with an increasing expression during B-cell differentiation. It is found only in late memory B cells and normal and malignant plasma cells at varying intensities (79–81). Therefore, B-cell depletion is not anticipated when targeting BCMA, as the majority of normal B-cells are BCMA_negative_. Although the toxicity profile of BCMA CAR T-cells seems to be manageable (80–82), alternative approaches have been developed to overcome this CAR T-cell therapy limitation.

### Antigen Escape: Down-Regulation or Loss of Target Antigen

Antigen escape post-infusion of modified CAR T-cells is an emerging issue (17, 83, 84). Relapses have been observed in long term follow-up studies, but the mechanisms for these relapses need to be better described. The most common reason for relapse after CAR T-cell therapy is the emergence of tumor cells with loss or downregulation of target antigen expression. The use of CD19 CAR T-cells in ALL patients has highlighted the loss of CD19 antigen expression due to therapeutic pressure. Consequently, CD19_negative_ tumor cells, have been reported in relapses. This can be caused by lymphoid to myeloid trans-differentiation, alternative splicing leading to target epitope loss, or selection of pre-existing antigen-negative leukemia cell clones (85–87). In the myeloma setting, BCMA loss or downregulation on residual MM cells after BCMA CAR T therapy has been reported in several clinical trials to date (29, 30, 88). Antigen escape in MM is more likely to occur due to the coexistence of numerous tumor subclones in treated patients resulting in a potential advantage to emerging or preexisting BCMA_low_ or BCMA_negative_ subclones during treatment (51). In the NCI trial, one patient presented persisting BCMA_negative_ myeloma cells in the bone marrow 56 weeks after BCMA CAR T-cell infusion and at the time of myeloma progression (29). In the UPENN trial, 12 out of 18 (67%) evaluable patients had significantly diminished levels of BCMA expression intensity commonly 1 month after CAR T-cell administration (30). This reduction in BCMA intensity was more frequent on residual myeloma cells from patients responding to therapy than non-responder patients. Regarding to FCARH143 trial, the authors reported a patient who relapsed presenting a BCMA_negative_ myeloma subclone and an overall reduction of 70% BCMA expression level in the BCMA_positive_ myeloma cell population (35). The observed loss and downregulation of BCMA expression on the surface of myeloma cells after CAR T-cell infusion in several clinical trials, highlights an imperative need to investigate the mechanism of resistance to BCMA CAR T-cell therapy.

### Soluble Protein: Hampering CAR T-Cell Function

A potential limitation for the clinical application of CAR engineered T-cells would be the presence of soluble protein antigens in the serum of patients. Target antigens can be cleaved from the cell membrane and released into blood circulation, therefore CAR T-cell therapy could be limited due to binding to soluble target antigens. Thus, the functionality of CAR T-cells could be abrogated by soluble antigens (89). In the myeloma setting, CD138 extracellular domain has been reported to be cleaved from cell surface, which could abrogate CAR T-cells function by blocking their antigen-binding domain and, hence, resulting in immune escape (90). Cleavage of the target antigen is not exclusive to CD138 protein, but it has been well-described that BCMA can be released from myeloma cells into the serum of patients. BCMA expression on the surface of myeloma cells can be modified by a protease called γ-secretase (GS), which mediates BCMA shedding within the transmembrane domain, leading to the release of a soluble fragment of BCMA (sBCMA) (91). Consequently, recognition of tumor cells by CAR T-cells could be hampered by soluble BCMA as a result of reducing BCMA density on the tumor cell surface and/or blocking the antigen-binding domain of the CAR. Thus, the presence of sBCMA in the serum of MM patients is being actively discussed as an obstacle for BCMA CAR T-cell therapy. In this sense, controversial data have been described. Several studies have reported that BCMA CAR T-cells were not abrogated by soluble BCMA protein (48, 80). However, alternative BCMA CAR T-cells have recently shown that sBCMA in concentrations of 10 ng/mL decreases the frequency of cytokine-producing BCMA CAR T-cells, at the same time that did not compromise CD19 CAR T-cells function. Moreover, high concentrations (333–1,000 ng/ml) of soluble BCMA protein affected cytotoxic capacity of CAR T-cells against 1 out of 3 BCMA_positive_ cell lines (92). These contradicting observations might be due to the use of different BCMA CAR T-cells directed to distinct epitopes, therefore it could happen that the BCMA-CAR target-epitope is not accessible in the soluble BCMA conformation.

### Quality of Harvested T-Cells: Insufficient Persistence of CAR T-Cells

Response rates of 64–85% have been achieved in myeloma patients treated with BCMA CAR T-cells (29, 30). However, only 8-39% of patients had a sustained VGPR or CR/sCR and this, together with the fact that most of the current clinical trials to date have reported BCMA_positive_ relapses, highlights a loss of efficacy of BCMA CAR T-cells against malignant plasma cells. This loss of efficacy might be a consequence of limited persistence of the CAR T-cells *in vivo*. Accordingly, long-term responses (>2 years) were unusually seen after BCMA CAR T-cell infusion (29–31). Nowadays, most of the CAR T-cell products are generated from autologous T-cells (17). Although this personalized cellular therapy has reported notable success in clinical trials (30, 31), the generation of CAR T-cells from patients could limit their application for multiple reasons. The first limiting factor is the harvesting of an adequate T-cell number from cancer patients who commonly present lymphopenia due to disease or previous treatments (17). Besides, the generation of autologous CAR T-cells might be a long procedure and progression of the disease could happen during manufacturing in advance-stage cancer patients (63, 93). Finally, intrinsic characteristics of apheresis products may be another limitation factor for the generation of autologous CAR T-cells (94). In the myeloma setting, harvesting the sufficient number of T-cells from patients seems to be feasible to produce CAR T-cells [e.g., the bb2121 clinical trial has shown that in 100% of patients who underwent leukapheresis, BCMA CAR T-cells were successfully generated (31)]. However, the quality of harvested T-cells in myeloma patients is likely a significant limiting factor due to deterioration of the immune system in these patients. Thus, the most solid indicator associated with response in the myeloma arena is the level of *in vivo* expansion and persistence of infused CAR T-cells (95). On the other hand, patient age (96, 97), number of prior lines of treatment (98), and the disease itself (99, 100) may limit the number and quality of patient-derived T-cells, potentially influencing the potency and variability of the CAR T products. Furthermore, the logistics of clinical manufacturing using patient-derived T-cells limits the accessibility of these therapies.

## Next Generation Car T-Cells in MM

**Figure 1:**
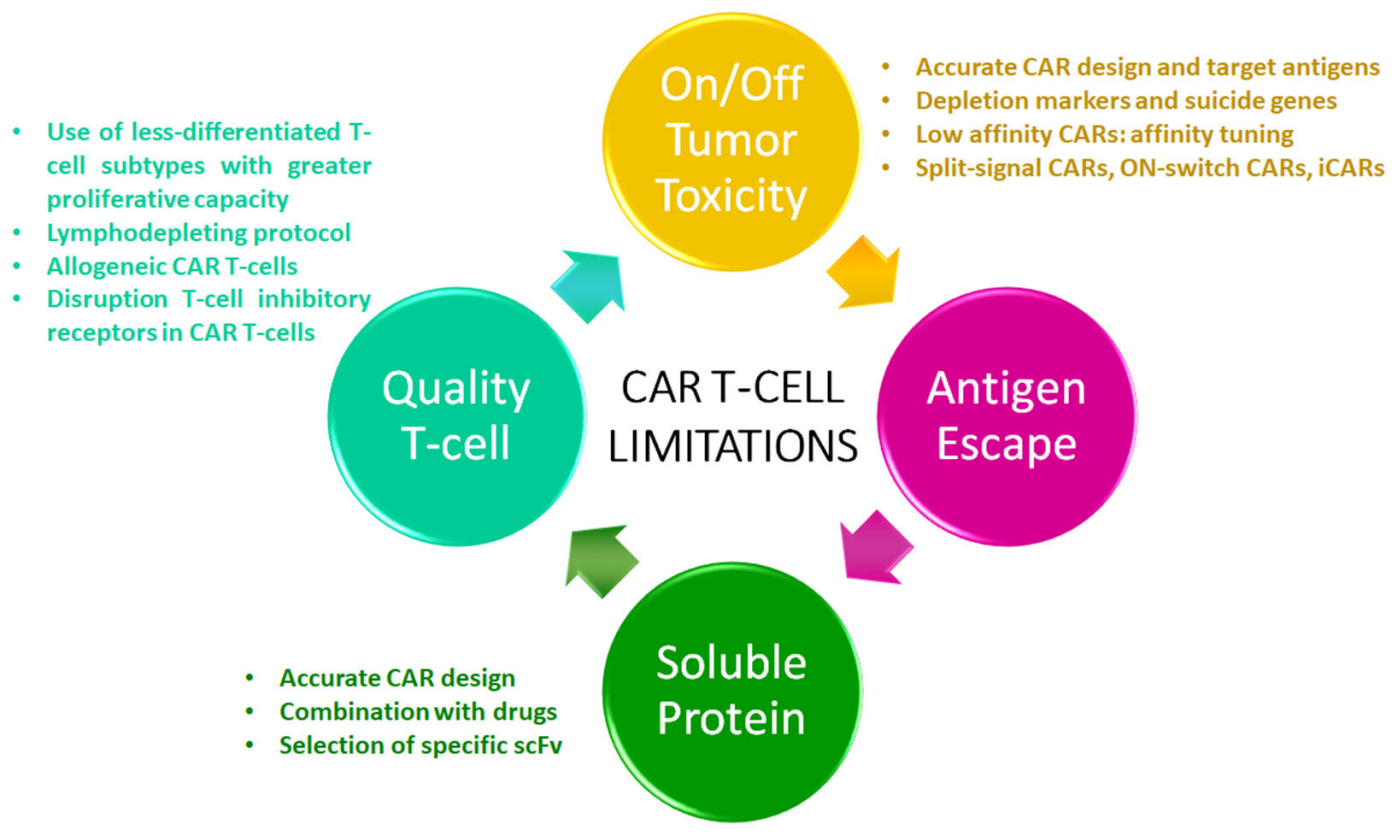
CAR T-cell limitations. The diagram shows the current limitations of this therapeutic modality in multiple myeloma. Strategies to overcome these limitations are listed.

To overcome CAR T therapy limitations in MM, new strategies have been developed in order to create next generation CAR T-cells to treat myeloma patients (Figure 1).

### Overcoming On/Off-Tumor Toxicities of CAR T-Cells

#### Overcoming On-Target, On-Tumor Toxicity

To achieve clinical efficacy while avoiding on-target, on-tumor toxicities, CAR T-cells activation and cytokine release must remain under a controlled level. The affinity of the antigen-binding domain for the tumor epitope, tumor burden, antigen density on the surface of cancer cells, and costimulatory domains present in the CAR, along with other factors, are implicated in the kinetics of CAR T-cell activation (101, 102). Dose-escalation schedules in phase I clinical trials are needed to determine the therapeutic window of CAR T-cell activation for each CAR due to differences in CAR design. In order to optimize this therapeutic window, diverse regions of the CAR gene can be accurately modified (17). In this sense, the costimulatory domain is considered one of the key points to optimize CAR design to reduce on-target, on-tumor toxicities. The most common costimulatory domains used in CAR T-cells are CD28 and 4-1BB. The use of CD28 costimulatory region has been correlated with a high and pronounced CAR T-cell activation and, hence, immune exhaustion phenotype. On the other hand, CAR T-cells with 4-1BB costimulatory region present lower peak of expansion, resulting in prolonged persistence and a lower risk of cytokine-mediated toxicities (103). Therefore, the costimulatory domain chosen in the CAR design may explain, at least in part, the toxicity-pattern differences observed in patients treated with CD28 containing CAR T-cells in which earlier onset CRS is more commonly observed as compared to patients treated with 4-1BB containing CAR T-cells (66). Some studies have also reported significant neurotoxicity and death from cerebral edema when CD28 containing CAR T-cell products were used (62). However, other clinical trials described no difference in the rate or grade of CRS and/or ICANS between CAR T-cells containing CD28 vs. 4-1BB costimulatory domains (63, 104, 105). Further studies are needed to better elucidate the link between costimulatory domain and toxicity events in patients receiving CAR T-cells. Others aspects of the CAR design such as the extracellular hinge region and/or transmembrane domain can also be optimized to reduce on-target, on-tumor toxicities. In fact, Ying et al. (86) generated a new anti-CD19 CAR variant [CD19-BBz (86)], which released lower levels of cytokines, expressed higher levels of anti-apoptotic molecules and proliferated more slowly than the prototype CD19-BBz CAR T-cells, while retained robust cytolytic activity. A phase I clinical trial was developed to evaluate this new variant and no significant CRS or ICANS events were reported while achieving a CR rate of 54.5% (106). In addition, novel strategies to control CAR T-cells toxicity include the engineering of depletion markers [e.g., truncated epidermal growth factor receptor [EGFRt]] and suicide genes (e.g., iCasp9) into the CAR design, providing a way to delete CAR T-cells if on-target, on-tumor (and/or on-target, off-tumor) toxicities appear. Accordingly, co-expression of the CAR gene together with the epitope recognized by clinically approved-monoclonal antibodies has been explored. For example, CD20 and EGFRt, which are targetable with rituximab and cetuximab, respectively (107–109). Furthermore, both depletion markers can be used to monitor T-cell transduction. Likewise, apoptosis of CAR T-cells can be induced by caspase pathway activation in iCasp9 next-generation CAR T-cells after the addition of the dimerization drug (109, 110).

#### Overcoming On-Target, Off-Tumor Toxicity

Multiple strategies to reduce off-tumor toxicities are now under development and are likely to offer novel clinically effective CAR T-cell products (111). Some of these approaches include the restriction of the recognition of normal cells by the CAR optimizing the specific interaction with tumor cells, either by (1) including the recognition of more restricted tumor-antigens or recognition of multiple antigens; and/or (2) limiting the spatial and temporal activity of CAR T-cells. In this sense, a novel target antigen has been reported for the immunotherapy of MM, GPRC5D. GPRC5D is a human orphan family C G protein-coupled receptor recently described to be expressed on 98% of CD138_positive_ cells (112, 113). The expression pattern of GPRC5D has proven to be very restricted in non-plasma tissues with the only exception of hair follicle cells. Consequently, GPRC5D CAR T-cells were generated by Smith et al. (113) showing anti-tumor efficacy against myeloma cells both *in vitro* and in a mouse model. Of note, GPRC5D CAR T-cells were also effective in eradication of myeloma after BCMA CAR T-cell treatment in a murine model which might be an option to overcome BCMA antigen escape. These data suggest that GPRC5D CAR T-cells could be an attractive alternative treatment in MM.

A distinct approach to limit off-tumor toxicities when a tumor-restricted antigen is not identified, could be the “affinity-tuning,” which is based on differences in density of antigen level among tumor and normal cells. Low-affinity CAR T-cells (low affinity scFv antibodies) could be generated to target antigens expressed at higher density on tumor cells than on normal cells. This is based upon published observations that T-cell activation initiated through the endogenous αβ TCR may result from reaching an activation threshold level, which can be triggered by binding a few high-affinity TCRs or greater number of low-affinity TCRs (114, 115). Several studies have demonstrated that a CAR with reduced affinity rendered T-cells preferentially activated by high, but not low, density of target antigen (116–119). However, immune escape has also been observed if downregulation of target antigen occurs (116, 120). In the myeloma setting, off-tumor toxicity profile of low affinity CD38 CAR T-cells has been evaluated. Drent et al. (71) showed that CD38 CAR T-cells with up to 1,000-fold decreased affinity were effective against CD38_positive_ myeloma cells, whereas CD38_positive_ healthy hematopoietic cells resulted unaffected.

Novel approaches to prevent off-tumor toxicities are the generation of CAR T-cells which require the recognition of two tumor antigens for their activation. In this regard, “split-CARs” co-expressing two different modules, one containing a scFv antibody along with CD3ζ (signal 1) and the second containing a different scFv antibody along with costimulatory domain (CD28, 4-1BB) have been explored (121, 122). This strategy may result in suboptimal CAR T-cell activation and limited off-tumor toxicity where only one antigen recognition occurs, which may be the case in normal cells. On the other hand, when both antigens are present, as in tumor cells, robust CAR T-cell activation is reached after double recognition (122). An alternative strategy consists of “ON-switch CARs” in which the antigen binding domain is dissociated from the signaling domain (CD3ζ), and CAR T-cell activation is controlled by a small molecule that induces dimerization (123). Subsequently, the intensity of CAR T-cells responses relies on the dose of dimerization molecule. Recently, “AND-gate CARs” has arisen as a promising technology. In this approach, the recognition of the first antigen by the synthetic Notch receptor induces the excision of the transcription factor which allows the expression of the CAR gene targeted against the second antigen. The expression of the CAR on the cell surface allows T-cell activation after antigen recognition (124). Therefore, CAR expression and CAR T-cell activation and hence, tumor elimination, can only happen when both target antigens are present. Nevertheless, the loss of the first antigen targeted by the synthetic Notch receptor which may result in immune escape, as well as slow activation kinetics, are the major limitations of this approach.

Another next-generation technology is the “inhibitory CAR” (iCAR) which incorporates the signaling region of an immunoinhibitory receptor (i.e., PD-1 or CTLA-4) to limit on-target, off-tumor toxicity. This novel strategy consists on the expression of a conventional CAR together with an iCAR in the same T-cell (125). The recognition of the target antigen by the iCAR restricts T-cell activation while the absence of this antigen allows CAR T-cell activation. Again, the challenge of this technology is finding antigens with an optimal expression pattern. First, iCAR should recognize antigens that are strictly expressed on normal cells whereas conventional CAR should recognize antigens that are specifically express on tumor cells.

### Overcoming Antigen Escape

**Figure 2:**
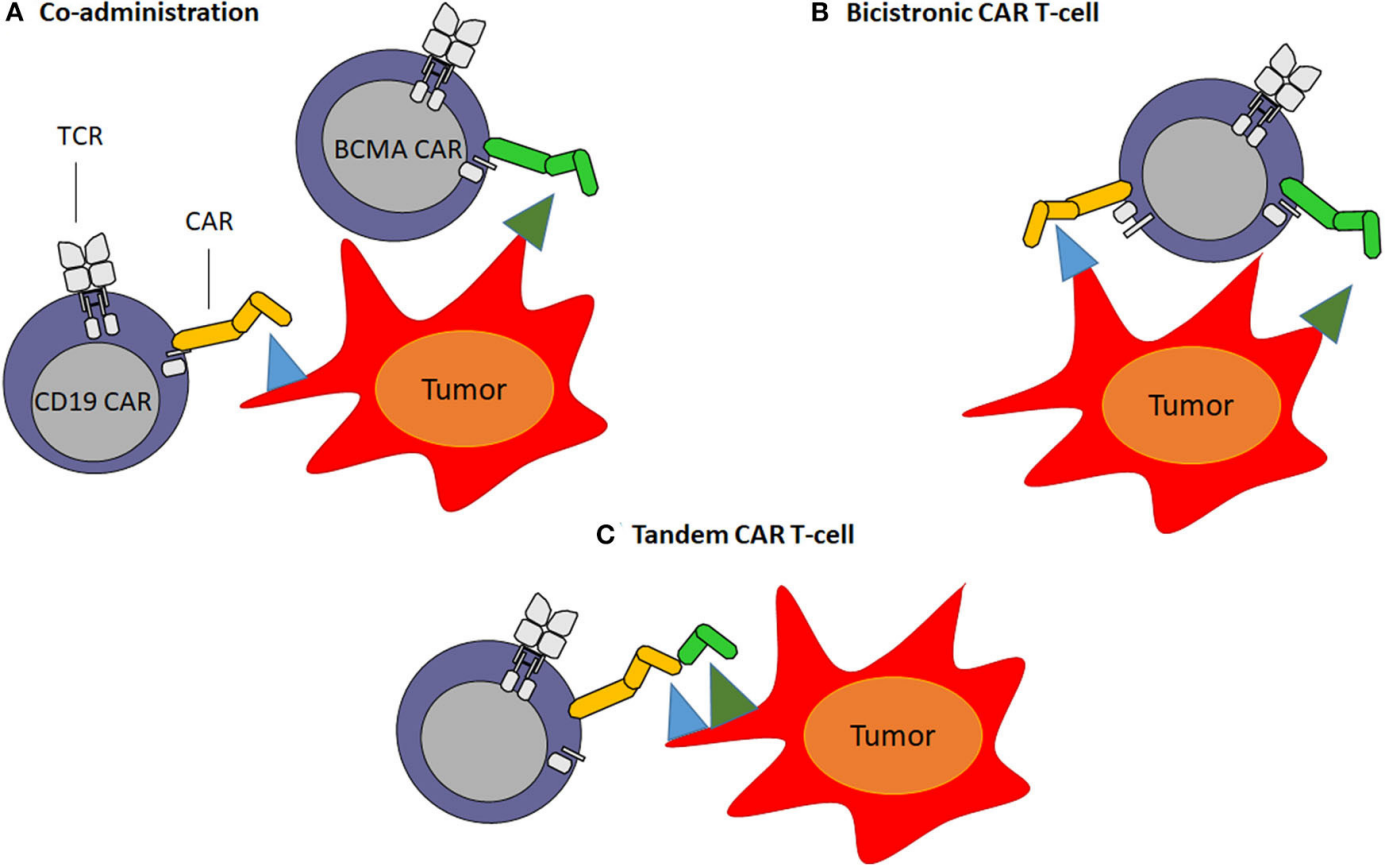
Multi-targeted CAR T-cell approaches. **(A)** Co-administration: involves production of two separate CAR T-cell products infused together or sequentially. **(B)** Bicistronic CAR T-cells: consist of the expression of two different CARs on the same T-cell. **(C)** Tandem CAR T-cells: consist of encoding two different scFv antibodies on same chimeric antigen receptor protein using a single vector.

CAR engineered T-cells targeting multiple antigens can be generated to address the antigen loss and therefore, reduce relapse rates (55). Next generation CAR T-cells can be developed by (1) combining different CAR T-cell products directed against single target antigens pre-infusion (co-administration of two or more different CAR T-cell products) (Figure 2A) or (2) transducing the same T-cell with two different complete CAR constructs (“bicistronic CAR T-cells”) (Figure 2B). Alternatively, bi-specific CAR T-cells, called also “tandem CAR T-cells,” can be generated by designing a single CAR construct with two (or more) different binding domains against two different antigens (126, 127) (Figure 2C). One important factor in the CAR design is the length of the transgene. In this sense, “tandem CAR T-cells,” which have smaller transgene length, show an advantage compared to “bicistronic CAR T-cells.” On the other hand, “tandem CAR T-cells” need design optimization including linker sequence, spacer size and VH-VL orientation between both scFv antibodies to accomplish desired antigens recognition and CAR T-cell activation (128). In 2019, Yan et al. (129) evaluated the efficacy and safety of the co-infusion of CD19 and BCMA CAR T-cells in relapse/refractory MM patients (NCT 03455972). CAR T-cell products were co-administrated on day 14–20 after autologous transplantation. The authors reported an ORR of 92.6% with 11/28 (40.7%) CRs or sCR, 8/28 (29.6%) VGPR and 5/28 (18.5%) PR. The median OS was 16 months. CRS was grade 1–2 in 19 (67.9%) patients (129). Although the exact mechanism of co-infusion remains unclear, expanding the coverage of MM cell targets might lead to better depletion of MM cell clonogenicity improving the duration of responses avoiding antigen escape. Co-infusion of two CAR T-cell products could likely reduce antigen escape, at the expense of a possible increase in toxicities as a result of simultaneous targeting of two or more antigens. Further studies with longer follow up and larger cohort of patients are needed in order to make conclusions regarding the use of combinatory CAR T-cell products. Another possible combination to overcome antigen escape limitation in MM is targeting both BCMA and TACI on myeloma cells. However, it has not been described whether BCMA-directed therapy affects TACI expression on the surface of residual myeloma cells. APRIL, a proliferation-inducing ligand, is the natural ligand of BCMA and TACI, and it is secreted in a trimeric form (130–132). In the preclinical setting, APRIL CAR T-cells have been successfully developed targeting both BCMA and TACI (133). Consequently, a clinical trial phase I/II is ongoing to evaluate the safety and efficacy of APRIL CAR T-cells in myeloma patients (NCT03287804) (134). Relapsed/refractory MM patients were infused with 15 × 10^6^ (1/11 pts), 75 × 10^6^ (3/11 pts), 225 × 10^6^ (3/11 pts), 600 × 10^6^ (3/11 pts), and 900 × 10^6^ (1/11 pts) APRIL CAR T-cells. The ORR was 43% (28 PRs and 14% VGPRs) in patients receiving ≥ 225 × 10^6^ CAR T-cells (134). Long-term follow up is needed to assess the efficacy of this dual-targeting CAR. Recently, Maus et al. (135) designed a novel human APRIL CAR T-cell product preserving its trimeric conformation (TriPRIL) showing enhance killing of both BCMA myeloma cell lines and primary myeloma cells. More recently, bicistronic BCMA/GPRC5D CAR T-cells are being explored and, preclinical data demonstrated that BCMA/GPRC5D CAR T-cells can limit BCMA antigen escape-mediated relapse in a murine model (136).

### Overcoming Abrogation of CAR T-Cells by Soluble Protein

Blocking the cleavage of target antigens from myeloma cells could be an interesting strategy to avoid the release of these target antigens as soluble proteins. In this sense, Pont et al. (92) have recently published that exposure to γ-secretase inhibitors (GSIs) efficiently blocks the release of BCMA from myeloma cells. Consequently, BCMA surface expression on MM cells is increased leading to a higher anti-tumor capacity of BCMA CAR T-cells, as well as improved cytokine production and proliferation in preclinical models (92). Relapsed myeloma patients with downregulated target antigen expression after BCMA-directed treatments could benefit from this novel GSI strategy. However, an optimal dose of GSI should be defined in order to avoid adverse effects on CAR T-cell function since the inhibition of GS did not impair viability or cytolytic activity but reduced IL2 production and proliferation of BCMA CAR T-cells (92). An alternative strategy might be the use of scFv antibodies to generate CAR T-cells directed against epitopes which are not accessible in the soluble protein conformation or belong to the extracellular region of the target protein that remains after cleavage. Thus, it has been reported that sBCMA folds and participates in the formation of high-molecular-weight complexes under physiological conditions (130, 137). Furthermore, data from three trials uncover the absence of correlation between sBCMA concentrations and extent of response (29, 56, 57). Therefore, the selection of the antigen-binding domain is a key factor in CAR design to overcome abrogation of CAR T-cells by soluble protein.

### Increasing CAR T-Cell Persistence

A promising approach to increase CAR T-cell persistence is the use of T-cell products containing a higher frequency of less-differentiated T-cell subtypes such as naïve T-cells (TN), stem cell memory T-cells (TSCM) and central memory T-cells (TCM), which have a superior proliferation capacity showing a delayed exhaustion or senescence immunophenotype (138–140). Compared with conventional CAR T-cell products, less-differentiated CAR T-cells have shown a greater proliferation and killing capacity in preclinical studies. To generate less-differentiated CAR T-cells, several strategies have been developed such as previous pre-selection of TN/TSCM subtypes or manufacturing in the presence of kinase inhibitors (138, 141, 142). In this sense, Shah et al. (37) designed a clinical trial with a next-generation CAR T-cell (bb21217) using the same construct as bb2121 but with a novel approach by employing phosphoinositide 3 kinase (PI3K) inhibitor bb007 during *ex vivo* expansion to enrich the CAR T-cell product in memory-like T-cells. To date, similar ORR and toxicity profile were observed in eight myeloma patients treated with the lowest dose of bb21217 (150 × 10^6^ CAR T-cells). However, longer follow-up is required. Clinical trials are currently ongoing using CAR T-cell products selectively generated from CD8+ TCM cells to evaluate safety and efficacy (NCT01087294) (142). Another strategy could be to define the CD4:CD8 ratio into the CAR T-cell product. In this sense, increased CAR T-cell expansion has been observed in patients after the infusion of a defined 1:1 (CD4:CD8) CAR T-cell ratio (35, 105, 143).

In most clinical trials conventional CAR T-cells derive from autologous T-cells. Generation of autologous CAR T-cells is a lengthy and elaborated process, time-consuming and logistically challenging, which comprises multiple steps including T-cell isolation and selection, transduction, expansion and infusion into patients. Along with that, the majority of patients are in relapsed/refractory stage and have already received numerous lines of toxic treatments, which further weaken T-cells quality and hence, reduce their immune response against tumor cells. Therefore, the quality/fitness of T-cells from which CAR T-cells are generated might also have an important role in CAR T-cell expansion and persistence, and anti-tumor capacity. An allogeneic CAR T-cell approach might have the potential to circumvent all these challenges by using healthy donor-derived T-cells to produce CAR T-cells which can be available as an off-the-shelf product (144–146). However, the use of allogeneic CAR T-cells could induce graft-versus-host disease (GVHD). In this context, host allo-antigens can be recognized via TCR by allogeneic CAR T-cells. On the other hand, the infused allogeneic CAR T-cells could be attacked by the host immune system owing to the HLA disparity resulting in CAR T-cell elimination. In consequence, further genetic modifications are needed in order to create next generation off-the-shelf allogeneic CAR T-cells. These genetic modifications consist of disrupting the endogenous TCR using gene-editing technology such as CRISPR/Cas9 and TALEN to limit the risk of GVHD, and the addition of a safety element to limit toxicities (14, 147, 148). Moreover, suppression of HLA class I expression by disrupting the HLA-A or b2-microglobulin genes is a novel strategy that is being evaluated to avoid allogeneic CAR T-cell elimination by the host immune system (149). Alternative approaches to prevent early allogeneic CAR T-cell rejection have been explored for instance, the use of more intensive lymphodepletion protocols. Nevertheless, the later immune system reconstitution of the lymphodepleted patient may be an obstacle for the allogenic CAR T-cell persistence (51). Advances in gene-editing techniques are leading to a new scenario in CAR T-cell therapy resulting in the development of universal CAR T-cells from allogeneic healthy donors. In this regard, preclinical results of allogenic second-generation BCMA CAR T-cells, called ALLO-715, were reported (150). In this study the authors, after CAR-transduction, transfected CAR T-cells with messenger RNA of TALEN. In particular, both CD52 and T-cell receptor α-chain genes were specifically disrupted resulting in CAR T-cell resistance to anti-CD52 lymphodepletion treatment, as well as preventing GvHD, respectively, without compromising CAR-mediated cytotoxicity. Moreover, this ALLO-715 BCMA-CAR incorporates a rituximab-sensitive safety switch. Currently, a phase I trial is ongoing to evaluate the safety and efficacy of the ALLO-715 product.

CRISPR/Cas9 gene-editing tool constitutes a promising technology to create next-generation CAR T-cells. This technology offers a wide range of CAR T-cell products, such as more potent CAR T-cells by disrupting inhibitory genes, allogeneic CAR T-cells by endogenous TCR and HLA elimination and novel CAR T-cells by knock-out of the targeted antigens to avoid fratricide effect. However, off-target toxicity is the major barrier of CRISPR/Cas9 gene-editing technology. These off-target toxicities can provoke non-desired deleterious consequences such as activating oncogenes or disrupting tumor-suppressor genes (151). To overcome this limitation, several approaches have been explored such as optimized sgRNA design and Cas9 activity, prior off-target detection assays, and careful selection of the target site to reduce off-target effects (152, 153). Therefore, a deeply understanding of the potential toxic effects of gene-editing in CAR T-cells is required.

Finally, to improve T-cell persistence and, hence T-cell response, we might target several T-cell inhibitory receptors such as PD-1, TIM-3, LAG-3, and CTLA-4 which send inhibitory signals into the T-cell. The expression of these inhibitory receptors on CAR T-cells leads to T-cell exhaustion. Recent studies show that tumor cells can take advantage of these T-cell inhibitory receptors in order to evade the immune response. For example, tumor cells upregulate PD-1 ligand (PD-L1) which causes reduced immune responses through PD1/PD-L1 pathway (154). Therapeutic strategies specifically designed to inhibit these inhibitory signals by immune checkpoint inhibitors, such as anti-PD-1 and anti-CTLA-4, have been described showing promising results in the treatment of solid tumors in addition to hematological malignancies (155, 156). This background has led to design directed-CRISPR/Cas9 technology in order to disrupt immune checkpoints. Some studies suggest an improvement in the anti-tumor efficacy and clinical outcome using these next generation modified CAR T-cells (157–159).

## Future Directions and Conclusions

CAR T-cell therapy is an outbreaking technology to treat hematologic cancers, however, several limitations need to be overcome in order to reach optimal patient response. Promising strategies have been proposed to optimize conventional CAR T-cells increasing safety and efficacy and improving manufacturing feasibility. Fundamental modifications in CAR design can be a promising strategy to reduce CAR T-cell toxicities. Although it is still not clear the influence of soluble antigens in CAR T-cell therapy, the existence of soluble proteins in serum of myeloma patients is being actively discussed as an obstacle for this immunotherapy. Therefore, the selection of the antigen-binding domain is a key factor in CAR design to overcome abrogation of CAR T-cells by soluble protein. In the myeloma setting, CAR T-cell treatment will probably become soon a key strategy for the treatment of relapsed/refractory patients. Likewise, resistance mechanisms to CAR T-cell therapy, such as antigen loss, need to be explored and strategies to overcome these limitations will be essential to ensure optimal efficacy in myeloma treatment. Development of novel strategies to increase long-term responses by combining CAR T-cell therapy with different drugs which increase antigen density specifically in myeloma cells avoiding antigen escape and toxicities is mandatory. Nowadays, CAR T-cells in myeloma are usually administered in patients who are refractory after numerous previous lines of treatment. Consequently, finding an adequate bridging therapy for these patients while CAR T-cell products are generated can be difficult. Can we obtain a better efficacy of CAR T-cells in earlier lines of treatment? Ongoing trials are looking at this question. Moreover, a new era is coming in CAR T-cell therapy in myeloma: allogeneic CAR T-cells derived from healthy donors can overcome some limitations as compared to conventional autologous CARs like manufacturing labor, production time and costs.

BCMA CAR T-cells have already proven safety and efficacy in relapsed/refractory patients and CAR T-cell therapy in myeloma is in great development with major efforts to optimize this approach all over the world. Even though there are more unsolved issues regarding the use of CAR T-cell therapy in MM, the field is still arising, and its full potential is about to discover.

## Author Contributions

EG-G wrote and revised the manuscript, figures and references, and supervised the tables. BS-M wrote the manuscript and tables, assisted in the elaboration of the references list. JP-S supervised the manuscript, figures and references.

## Conflict of Interest

EG-G is an inventor on a patent related to chimeric antigen receptor technologies in myeloma that have been filed by the University of Wurzburg (Wurzburg, Germany). The remaining authors declare that the research was conducted in the absence of any commercial or financial relationships that could be construed as a potential conflict of interest.
